# Impacts of heatwaves on type 2 diabetes mortality in China: a comparative analysis between coastal and inland cities

**DOI:** 10.1007/s00484-024-02638-0

**Published:** 2024-02-26

**Authors:** Wenxiu Zheng, Jie Chu, Hilary Bambrick, Ning Wang, Kerrie Mengersen, Xiaolei Guo, Wenbiao Hu

**Affiliations:** 1 Ecosystem Change and Population Health Research Group, School of Public Health and Social Work, Queensland University of Technology, Brisbane, QLD 4059 Australia; 2 Shandong Center for Disease Control and Prevention, and Academy of Preventive Medicine, Shandong University, Jinan, Shandong China; 3 National Centre for Epidemiology and Population Health, Australian National University, Canberra, Australian Capital Territory Australia; 4 National Center for Chronic and Noncommunicable Disease Control and Prevention, Chinese Center for Disease Control and Prevention, Beijing, China; 5 School of Mathematical Sciences, Queensland University of Technology, Brisbane, QLD Australia; 6 Centre for Data Science, Queensland University of Technology, Brisbane, QLD Australia

**Keywords:** Heatwave, T2DM, Inland, Coastal, Shandong

## Abstract

The impacts of extreme temperatures on diabetes have been explored in previous studies. However, it is unknown whether the impacts of heatwaves appear variations between inland and coastal regions. This study aims to quantify the associations between heat exposure and type 2 diabetes mellitus (T2DM) deaths in two cities with different climate features in Shandong Province, China. We used a case-crossover design by quasi-Poisson generalized additive regression with a distributed lag model with lag 2 weeks, controlling for relative humidity, the concentration of air pollution particles with a diameter of 2.5 µm or less (PM_2.5_), and seasonality. The wet- bulb temperature (Tw) was used to measure the heat stress of the heatwaves. A significant association between heatwaves and T2DM deaths was only found in the coastal city (Qingdao) at the lag of 2 weeks at the lowest Tw = 14℃ (relative risk (RR) = 1.49, 95% confidence interval (CI): 1.11–2.02; women: RR = 1.51, 95% CI: 1.02–2.24; elderly: RR = 1.50, 95% CI: 1.08–2.09). The lag-specific effects were significant associated with Tw at lag of 1 week at the lowest Tw = 14℃ (RR = 1.14, 95% CI: 1.03–1.26; women: RR = 1.15, 95% CI: 1.01–1.31; elderly: RR = 1.15, 95% CI: 1.03–1.28). However, no significant association was found in Jian city. The research suggested that Tw was significantly associated with T2DM mortality in the coastal city during heatwaves on T2DM mortality. Future strategies should be implemented with considering socio-environmental contexts in regions.

**Supplementary Information:**

The online version contains supplementary material available at 10.1007/s00484-024-02638-0.

## Introduction

An ongoing threat to human health worldwide, diabetes has resulted in 6.7 million deaths in 2021, and 643 million adults are predicted to be living with diabetes by 2030 (IDF 2023). The long-term cost of care and the necessity for strict adherence to multiple medications pose a considerable burden on individuals living with diabetes (Rodbard et al. 2010). Type 2 diabetes mellitus (T2DM) accounts for the significant majority of diabetes cases, around 90.0% of overall diabetes cases in China (Society CD 2010) and 96.0% of diabetes cases worldwide (GBD 2023). Furthermore, in mainland China, the latest national survey in 2017 reported that around 11.2% of adults had diabetes (90% being T2DM), which was much higher than the global average prevalence (8.8%) (Li et al. 2020).

The Intergovernmental Panel on Climate Change predicts that global warming will intensify due to rising cumulative CO_2_ emissions, highlighting an increase in the frequency of heatwaves (IPCC 2023). It is highly likely that the health burden associated with heatwaves will escalate along with climate change. Previous studies have demonstrated the detrimental impact of heatwaves on diabetes mortality and hospitalization. For example, in a time series study conducted across multiple cities in China, the combined relative risks (RR) of the 90th percentile temperature on diabetes mortality were found to be 1.11 (95% confidence interval (CI): 1.03–1.19) at a lag of three weeks, in comparison to the 75th percentile of temperature (Yang et al. 2016). In a multi-city study conducted in Brazil, it was observed that for every 5 °C rise in the daily mean temperature, there was a 6% increase (RR = 1.06, 95% CI: 1.04–1.07) in hospitalizations caused by diabetes (Xu et al. 2019). In addition, combined with the occurrence of heat, high humidity can aggravate the adverse effects of heat on human body (Davis et al. 2016). The humid and warm environment could restrict the evaporation process, leading to higher risks of mortality (Parsons 2019). The wet-bulb temperature (Tw) comprehensively characterizes the temperature and humidity (Stull 2011), and has been used as an efficient index of heat stress in previous studies (Freychet et al. 2020; Yu 2021). However, these national studies did not further explore the potential for regional disparity in the effects.

In our prior spatial study of diabetes mortality in Shandong Province, China, we identified two significant clusters of diabetes mortality, corresponding to inland and coastal areas in the province (Zheng 2022), and different impacts of environmental factors (e.g., temperature, relative humidity) were identified between the inland and coastal areas (Zheng 2023). In recent years, studies have started to highlight the potential effects of different environmental contexts between inland and coastal areas on disease burdens. The comparative analysis of disease burden between inland and coastal areas has been conducted for several diseases, such as inflammatory bowel disease, malaria and cardiovascular diseases (Carpio et al. 2015; Miyashita et al. 2015; Nyasa et al. 2022). However, to the best of our knowledge, previous studies investigating the impacts of the ambient environment on diabetes widely included all types of diabetes without specifically targeting T2DM. Given that T2DM is more likely to be preventable by adjusting social and environmental factors compared to type 1 diabetes (WHO 2023). In addition, the potential different impacts of heatwaves on the diabetes mortality between the inland and coastal areas has not specifically explored. Therefore, there is a need for further investigation to elucidate the specific impacts of environmental factors on T2DM, particularly in relation to heatwaves in different regions.

The case-crossover design proposed by Maclure effectively controls for time-invariant confounders in subjects at both the event date and the reference time (Maclure 1991), such as demographic characteristics (e.g., age, gender, socioeconomic status) and local regional factors, as well as the potential bias from seasonality (Janes et al. 2005). However, the potential delayed and non-cumulative effects of the risk factors cannot be precisely and directly measured by case-crossover design alone. Regarding this limitation, DLNM has been widely used to estimate the non-linear and lagged effects of risk factors on health outcomes simultaneously, providing a more comprehensive understanding of the associations between variables (Armstrong 2006). In the framework, ‘cross-basis’ generates a two-dimensional risk-response relationship, encompassing both immediate and lag dimensions (Armstrong 2006). Thus, both cumulative effects over the period and lag-specific effects can be evaluated in the model by evaluating contributions at different lag times, considering the lagged effects and short-term health outcome displacement (Cox et al. 2016). Therefore, in this study, the combination of DLNM with the case-crossover design was employed to evaluate the short-term, non-linear and lagged effect of heatwaves with controlling for time-invariant confounders and long-term trend, providing a more precise examination of exposure–response associations (Fu et al. 2018; Guo et al. 2011; Pan et al. 2022; Wang et al. 2020).

This study first examined the disparity in the impact of heatwaves on T2DM deaths between inland and coastal areas. Jinan and Qingdao, the two big cities in Shandong Province, were selected as typical inland and coastal study sites respectively in this study. The associations between heat exposure and T2DM deaths were calculated by the combination of the distributed lag non-linear model (DLNM) and case-crossover design. The findings will provide valuable insights for the development of better-targeted strategies to deal with the challenges of escalating extreme weather events resulting from anthropogenic global warming, such as the effective and more efficient allocation of resources for protective measures in the future.

## Methods

### Study sites

With a population of 101 million residents, Shandong Province ranks as the second most populous province in China (Shandong Provincial Bureau of Statistics 2022). Jinan (36°65′ N, 117°00′ E) is the capital city of Shandong, covering 8,177 km^2^ area with over 9.3 million population, and Qingdao (36°06′ N, 120°38′ E) is the well-developed coastal city, covering 11,293 km^2^ area with over 10 million population (Shandong Provincial Bureau of Statistics 2022). Socioeconomically they are also similar, with Gross Domestic Product ranking as the top two across the whole province (Shandong Provincial Bureau of Statistics 2022). However, in terms of climate, Jinan is characterized by a warm temperate continental monsoon climate, whereas Qingdao has a distinct maritime climate (temperate oceanic monsoon zone) as Qingdao's urban areas are directly influenced by the marine environment (China Meteorological Administration 2014; Jinan Municipal Government 2022) (Fig. S1). Thus, the similarities in demographic and socio-economic characteristics, but with different prevailing climates, enable the comparison of environmental impacts between the two cities.

### Data collection

T2DM death cases were obtained from the National Death Surveillance System of China between 1st January 2013 and 31st December 2019, using ICD–10 codes (E11: Type 2 Diabetes Mellitus) (WHO 2016). Population data was collected from the Statistical Yearbooks during the study period (Shandong Provincial Bureau of Statistics 2019). Daily concentrations of PM_2.5_ were derived from the global reanalysis dataset provided by the Copernicus Atmospheric Monitoring Service (Inness et al. 2019). Daily temperatures and relative humidity data were collected from the National Oceanic and Atmospheric Administration (NOAA/OAR/ESRL PSL 2022). The data analysed for this study was specifically limited to the summer season, from 1st June to 31st August each year.

### Statistical analysis

#### Heatwave index

The heatwave stress was measured by Tw in this study. Weekly mean temperatures and relative humidities during the summers (1st June to 31st August) from 2013 to 2019 were calculated. Following the equation proposed by Stull (Stull 2011), we calculated the Tw as:

$$Tw=T arctan\left[0.151977 {\left(RH\%+8.313659\right)}^\frac{1}{2}\right]+arctan \left(T+RH\%\right)-arctan \left(RH\% -1.676331\right)+0.00391838 {\left(RH\%\right)}^\frac{3}{2} arctan \left(0.023101RH\%\right)-4.686035$$

where Tw is the wet-bulb temperature (℃), T is weekly mean temperature (℃) and RH is relative humidity (%).

#### Spatial analysis

Spatial interpolation of weekly death mortality rates across the entire Shandong Province was performed using the ordinary kriging method, utilizing data from national surveillance counties (Cressie 1988). Subsequently, city-level mortality rates for Jinan and Qingdao were derived through zonal function using city shapefiles in ArcGIS software (version 10.8). The detailed method has been described in our previous study (Zheng 2023). The number of death cases was then calculated by the interpolated city-level rates and population data.

#### Regression analysis

To assess the relationships between T2DM deaths and heatwave exposure days, this study utilized a time-stratified case-crossover design incorporating the DLNM (Armstrong et al. 2014; Gasparrini et al. 2010; Guo et al. 2011). To address the overdispersion in the distribution of T2DM death cases, a quasi-Poisson link function was employed in R software (Gasparrini 2011). The case date was defined as the number of weeks of the date of death. The referent dates were matched on the same weeks in other years during the study period. We used a DLNM combining with the case-crossover design as follows (Armstrong et al. 2014; Gasparrini 2011):

$${Y}_{t}\sim quasi-Poisson \left({\mu }_{t}\right)$$

$$\begin{array}{c}{\text{log}}\left[E\left({Y}_{t}\right)\right]={\beta }_{0}+now+cb\left({Tw}_{ld},df=3\right)+ns\left({PM}_{2.5},3\right)+ns\left(time,df=4\right),\\ elimanate (stratum), offset({\text{log}}\left(pop\right))\end{array}$$

where *Y*_*t*_ is the reported number of deaths in week t; *E(Y*_*t*_*)* is the expected death count in week t; *β*_*0*_ is the residual of the model; *now* is the number of the week in that year; *cb* is the cross-basis function fitting both the exposure (non-lag)- and lag-response relationships; *Tw* is the weekly wet-bulb temperature; PM_2.5_ is the weekly concentrations of PM_2.5_; *ld* is the lag weeks; *df* is the degrees of freedom; *ns* is the natural cubic spline with *df* = 3; *time* is the variable to control for the long-term trends; *stratum* is a categorical variable of the number of the week in the year to control for time-series trends; *pop* is the corresponding population data. We designed the lag up to two weeks as we considered the results of cross-correlation (1 week) and a literature review about the heatwave effects on diabetes suggested the lag time for studies to be more than 10 days (Moon 2021). The optimal model was chosen by the minimum value of the quasi-Akaike information criterion (QAIC) (Gasparrini et al. 2010; Peng et al. 2006). The model selection criterion to gain widespread acceptance was the Akaike information criterion (AIC) (Bozdogan 1987), and for quasi-Poisson distribution data, the modified AIC, QAIC is used to choose the best model (Anderson et al. 1994).

### Sensitivity analysis

To ensure the robustness of the models and examine potential biases from additional factors, we conducted tests by adding the variable of ‘Holiday’, as 'Holiday' may potentially play a role in the effect of heatwaves as indicated in the previous study (Guo et al. 2011). The reliability of the estimated data of T2DM deaths generated using the kriging method was evaluated by comparing it with a different death surveillance dataset collected by the Shandong Center for Disease Control and Prevention (CDC) from 2013 to 2019.

Associations were assessed using RR with 95% CI. Statistical significance was determined when the two-tailed *p* < 0.05. The 'dlnm' package in R software (version 4.2.2) was employed for statistical analyses in this study (Armstrong et al. 2014; Gasparrini 2011).

## Results

### Descriptive analysis

**Fig. 1:**
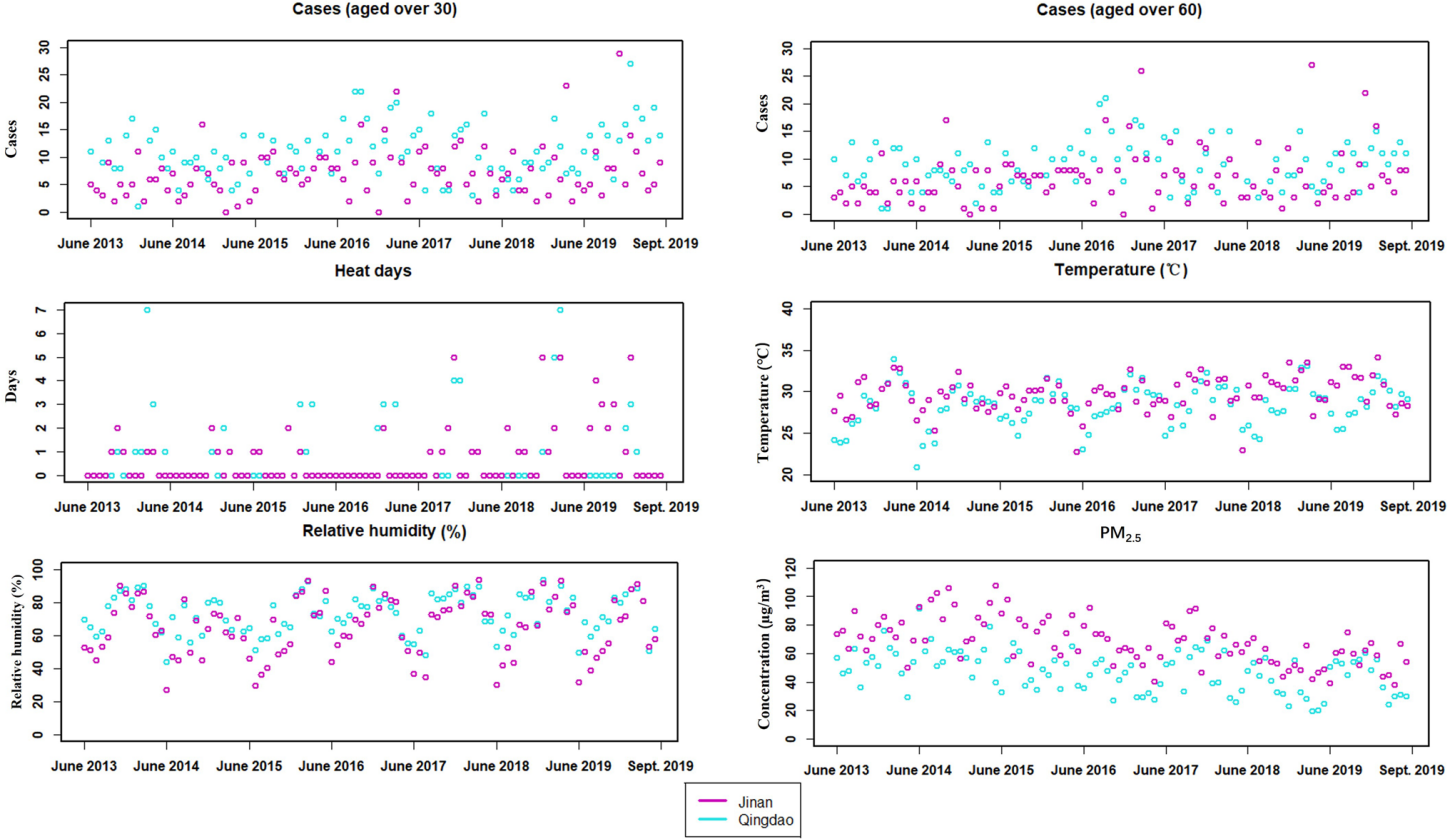
Time-series distribution of T2DM death cases and environmental factors in two cities, summer, 2013‒2019

Figure 1 illustrates the temporal distribution of weekly T2DM death counts and environmental factors over the study period (Fig. 1). It is evident that Qingdao had a higher number of cases during the summer months between 2013 and 2019 compared to Jinan. Although heatwaves occurred earlier in Jinan, Qingdao experienced a greater total number of heatwave days. In addition, Jinan had a significantly higher weekly average PM_2.5_ concentration compared to Qingdao. Furthermore, Qingdao, as a coastal city, demonstrated notably higher weekly mean relative humidity in comparison to Jinan, the inland city. Table 1 shows similar heatwave characteristics with city-specific thresholds respectively. Table 2 presents the characteristics of Tw in Jinan and Qingdao. The inland city (Jinan) had higher minimum Tw than the coastal city (Qingdao). Table 3 shows consistent distribution characteristics among subgroups, where the number of cases in women and elderly groups was higher compared to other subgroups, despite Qingdao having more total cases than Jinan.

**Table 1:** Summary statistics of the heatwaves in two cities, 2013‒2019

	Qingdao		Jinan	
	Temp (℃)	Heat days (weeks)	Temp (℃)	Heat days (weeks)
Summer				
90th	32.40	66 (28 w)	33.51	65 (33 w)
95th	33.09	33 (18 w)	34.07	35 (20w)

Days (total number of weeks with heatwaves during the entire observation period)

**Table 2:** Characteristics of heat stress index (Tw)

City	Min	Median	Max	SD
Qingdao	13.65	24.77	32.50	3.61
Jinan	15.18	24.63	31.06	3.51

Min, minimum; Max, maximum; SD, standard deviation

**Table 3:** Summary statistics of the T2DM death cases in two cities, 2013‒2019

Weekly death cases		Qingdao				Jinan			
		Median	Min	Max	SD	Median	Min	Max	SD
Total	Adults (> 30 years old)	10.88	1	27	4.94	7.31	0	29	4.71
Gender									
	Men	4.86	0	17	2.92	3.59	0	16	2.94
	Women	6.32	1	16	3.37	4.2	0	19	3.17
Age									
	30‒60 years	1.48	0	7	1.59	1.23	0	8	1.34
	60‒ years	8.71	1	21	4.17	6.73	0	27	4.95

SD, standard deviation; Min, minimum; Max, maximum

### Association between heatwaves and T2DM mortality in coastal city ‒ Qingdao

**Fig. 2:**
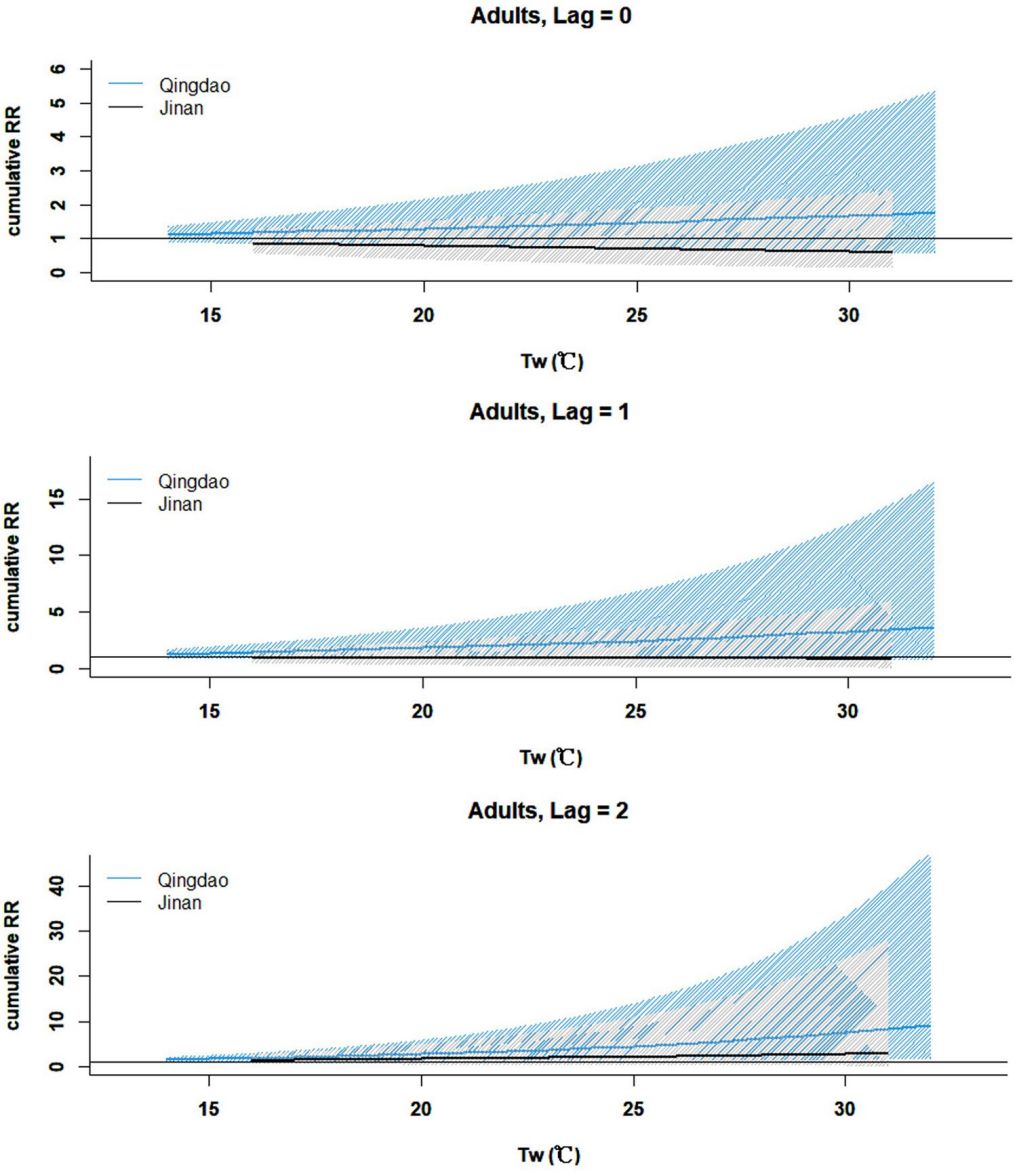
Cumulative associations (RR and 95% CI) between heatwaves and T2DM death cases for adults in Jinan and Qingdao

**Fig. 3:**
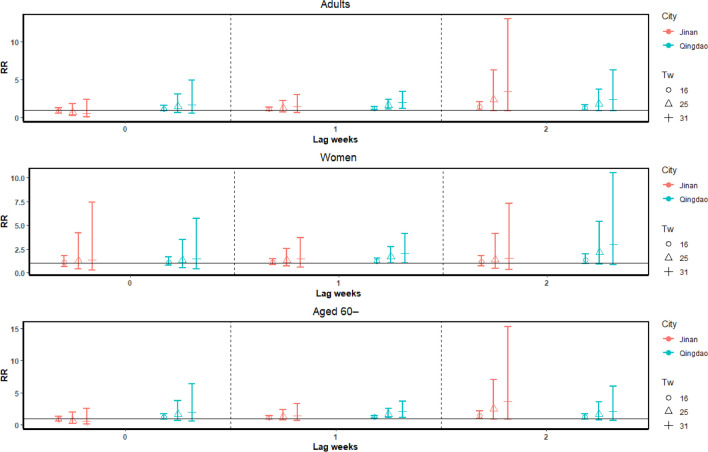
Lag-specific associations between heatwave days and T2DM death cases in Jinan and Qingdao

Significant increases in T2DM deaths were observed across all adults, women, and elderly (aged over 60) groups. Tw ranged from 14℃ to 32℃ in Qingdao. The cumulative associations are significant at the lowest Tw = 14℃ at the lag of 2 weeks (RR = 1.49, 95% CI: 1.11–2.02; women: RR = 1.51, 95% CI: 1.02–2.24; elderly: RR = 1.50, 95% CI: 1.08–2.09). The impacts of heat stress accumulated over time, and peaked at 2 weeks (Fig. 2). The lag-specific pattern depicted in Fig. 3 indicated an increase specific association from week 0 to week 1, and then decreased from week 1 to week 2, with peaking associations at lag 1 week. The most notable lag-specific associations were observed at the lowest Tw = 14℃ at the lag of 1 week (RR = 1.14, 95% CI: 1.03–1.26; women: RR = 1.15, 95% CI: 1.01–1.31; elderly: RR = 1.15, 95% CI: 1.03–1.28). In order to show the comparison between Jinan and Qingdao, Fig. 3 presented the associations at the key Tw values in these two cities (minimum (16℃), median (25℃) and maximum (31℃) values). Both the trend of increasing of Tw values and the trend over time were showed in Fig. 3. As observed in the cumulative and lag-specific results (Figs. 2 & 3), the mortality risks displayed a significant upward trend as the increase of heat stress in a week.

### Association between heatwaves and T2DM mortality in the inland city ‒ Jinan

No statistically significant results were found in all subgroups of residents in Jinan. The heat stress index did not show significant associations with the T2DM mortality in the inland city. As illustrated in Fig. 2, the temperature range of Tw in the inland city was observed to be smaller than the coastal city. In line with the trend in the coastal city, the risks of T2DM mortality in inland city exhibited an upward trend of the cumulative effects from lag 0 to lag 2 weeks (Fig. 2). Moreover, with the increasing Tw values, there was a corresponding increase in the risks of T2DM mortality in the inland city, as depicted in Fig. 0.3.

### Comparison between Qingdao and Jinan

Qingdao exhibited greater vulnerability to heatwaves, with rapid responses observed to heatwaves in total adults (aged over 30 years old), as well as specific women and elderly (aged over 60 years old) subgroups. In contrast, Jinan did not display significant responses to heatwaves in any subgroups. Moreover, gender‒specific vulnerable subgroups were identified, with women in Qingdao being particularly susceptible to heatwave exposure. And the adverse effects increased with the increasing of heat stress.

### Sensitivity test results

Adding ‘Holiday’ in the model led to higher QAIC values (Table S1). Diabetes death data from the dataset collected by Shandong CDC across the Shandong Province showed similar trends to our estimated data, demonstrating the reliability of the results in this study (Fig. S2).

## Discussion

The lag effects of heatwave exposure on T2DM deaths were examined in this study in an inland city and a coastal city in Shandong Province, which is characterized as a province with a large population across China. To the best of our knowledge, this is the first study that used DLNM with combining the case-crossover design simultaneously in studies of diabetes at different climate features in China. The modeling approach utilized in this research can serve as a valuable reference for future studies on climate-related health effects.

Previous studies on health have found that the distinct inland and coastal socio-environmental factors led to varying impacts on the risk of diseases. Consistent with our previous study, diabetes deaths in coastal areas were more sensitive to temperatures than inland areas, with women group being the vulnerable population (Zheng 2023). This study confirmed the heat effects and further identified the combined effects with higher humidity during heatwaves on diabetes deaths. Living in coastal areas could increase the prevalence risk of eczema symptoms in children compared to living in the inland areas (Miyashita et al. 2015). For ulcerative colitis, living in coastal areas was turned to be a protective factor (Carpio et al. 2015). And regarding the hospital admissions of cardiovascular diseases in Shandong Province, air pollution had a more pronounced negative impact on the overall cardiovascular disease exacerbations in the inland city (Jinan) than in the coastal city (Weihai), as well as different invariable subgroups (Liu 2020). In this study, the spatial heterogeneity in heatwave-related T2DM deaths between inland and coastal areas was also identified by robust time series statistical models.

Diabetes deaths in this inland city did not show a significant increase during heatwaves, including in different age and gender subgroups. In contrast, the coastal city of Qingdao exhibited a significant response to heatwaves. This may potentially be attributed to the relatively higher temperature value in Jinan than in Qingdao, prompting residents to implement strategies like earlier and more frequent use of air conditioning to mitigate the impact of high temperatures as the earlier and longer period for heatwaves regularly in summers in Jinan (Chung et al. 2018; Reid et al. 2009). Another plausible explanation is that long-term exposure to higher temperatures in Jinan may have led to the development of increased tolerance and adaptability among the local population. For example, a literature review indicated that population adaption to heat in recent decades led to a decrease in susceptibility to heat, and the extent of the decrease varied by location (Arbuthnott et al. 2016). Also in a multi-country investigation of temporal trends in heat-mortality impacts, some levels of adaptation to heat were found to be prevalent, so adaptation might be a consideration here, limiting the impacts of heat (Vicedo-Cabrera et al. 2018).

The risks of T2DM mortality increased with the increasing of Tw values both in the inland and coastal city in this study, although only significant in the coastal city. This further confirms that the adverse impacts on health could increase with the increasing intensity of heat weather (Xu et al. 2017; Tong et al. 2015). As Tw included the impacts of humidity, the disparity in the impacts of heatwaves on the T2DM mortality between inland and coastal cities reflected the role of relative humidity during the heatwaves. Consistent with the previous studies, higher humidities could aggravate the adverse effects of solely higher temperatures, which further provided evidence for the necessity of considering humidity in studies assessing the impacts of heatwaves (Al-Qaissi et al. 2019; Davis et al. 2016; Tyrovolas et al. 2014). The higher relative humidity in Qingdao led to higher heat stress, Tw values, during the heatwaves, which might be a possible reason for the higher adverse impacts on T2DM mortality in the coastal areas in this study. Therefore, Tw was highly recommended to be applied in the explorations.

Regarding the biological evidence for the adverse impacts of hot weather on diabetes patients, a study suggested that the disturbances in autonomic regulation during periods of elevated temperatures could be a driving factor behind the fluctuations in apparent glucose tolerance among individuals living with diabetes (Moses et al. 1997). Additionally, during hot weather, there may be a potential allocation of blood flow between the skin and internal organs, which could have an impact on glucose tolerance (Forst et al. 2006; Zanobetti et al. 2014). A previous study also provided evidence that changes in insulin absorption during hot weather could contribute to variations in glucose tolerance (Koivisto et al. 1981).

The gender-specific impacts were further explored in this analysis. Women in Qingdao were vulnerable subgroups with statistically significant responses to heatwaves. Previous multi-city studies have seldom investigated the discrepancies among subgroups, typically pooling the risks by meta-analysis to reach the general conclusion for that region (He et al. 2020; Huang et al. 2018; Yang et al. 2019). This limitation arises from variations in socio-economic factors and population density across the different study sites. This study reveals that population sensitivity to heatwaves may vary among different cities, even within the same province. The exact reasons for these variations remain unclear. Further research is needed to determine the factors contributing to the susceptibility of these subgroups, such as the differences in occupation between genders in cities, which is essential for designing targeted intervention plans (Shockey et al. 2017).

The elderly population (aged over 60) consistently exhibited the highest vulnerability in coastal areas, aligning with the characteristic pattern of type 2 diabetes mellitus (T2DM) where prevalence increases with age. This conclusion is consistent with previous studies about the heat response of diabetes (Bai et al. 2016; Xu et al. 2019; Yang et al. 2019).

This study had notable strengths in first investigating the T2DM death risks of both inland and coastal populations exposed to heatwaves. It uses data from a recent time period, spanning from 2013 to 2019, with a seven-year period and a large sample size, allowing for a robust evaluation of the association between T2DM deaths and heatwaves. Additionally, we observed a change in the frequency of heatwave days in each year between the two areas, with Jinan experiencing an increase in heatwave days starting from 2018, contrary to previous years (Fig. S4). However, the limitations of this study should also be noted. First, as an ecological analysis, risk factors at the individual level could not be controlled, and ecological bias existed. In this study, we estimated mortalities in areas not covered by the surveillance system using kriging interpolation, which may influence the evaluation. The surveillance points within and around the city might affect the kriging interpolation results, as the kriging process considered the data in the neighbor areas. However, the estimated data showed similar trends and patterns to reported data. Second, the misclassification of diabetes cause of death in the data collection process cannot be ignored, particularly when deaths are attributed to cardiovascular diseases which are the complications of diabetes. Third, during heatwave days, people tend to decrease outdoor activities, which may underestimate the adverse impacts of heatwaves. It is also insufficient to extend this conclusion to other regions directly. Further studies are recommended to include more similar cities and consider additional potential confounders, such as demographics and work intensities.

## Conclusions

The findings of this study revealed distinct responses to different intensities of heatwaves between the inland city (Jinan) and the coastal city (Qingdao) in Shandong Province. Although Jinan had relatively higher mean temperatures in the summer period than Qingdao, it showed less sensitivity to heatwaves, which highlighted the importance of considering the role of the combination impacts of temperatures and relative humidity, especially in summers. This emphasizes the significance of considering long-term living conditions and residents' adaptability when implementing strategies to mitigate the adverse effects of heatwaves.

### Supplementary Information

Below is the link to the electronic supplementary material.

Supplementary file1 (DOCX 623 kb)

## Data Availability

Data are only available on reasonable request and approved by the Center for Chronic and Non-communicable Disease Control and Prevention, Shandong Center for Disease Control and Prevention.
