# Supplementary material for: Impacts of heatwaves on type 2 diabetes mortality in China: a comparative analysis between coastal and inland cities
Source: Int J Biometeorol. 2024 Feb 26;68(5):939–48. doi: 10.1007/s00484-024-02638-0 (PMC11058751; doi:10.1007/s00484-024-02638-0)
Supplement: Supplementary file 1 — Supplementary file1 (DOCX 623 kb) [file 484_2024_2638_MOESM1_ESM.docx]

Supplementary materials

**Impacts of heatwaves on type 2 diabetes mortality in China: A comparative analysis between coastal and inland cities**

**Authors**

Wenxiu Zheng^a^, Jie Chu^b^, Hilary Bambrick^a,c^, Ning Wang^d^, Kerrie Mengersen^e^, Xiaolei Guo^b^, Wenbiao Hu^a,*^

**Author affiliations**

^a^Ecosystem Change and Population Health Research Group, School of Public Health and Social Work, Queensland University of Technology, Brisbane, Queensland, Australia

^b^Shandong Center for Disease Control and Prevention, and Academy of Preventive Medicine, Shandong University, Jinan, Shandong, China

^c^National Centre for Epidemiology and Population Health, Australian National University, Canberra, Australian Capital Territory, Australia

^d^National Center for Chronic and Noncommunicable Disease Control and Prevention, Chinese Center for Disease Control and Prevention, Beijing, China

^e^School of Mathematical Sciences, Queensland University of Technology, Brisbane, Queensland, Australia

**Corresponding author**

*Prof. Wenbiao Hu, Ecosystem Change and Population Health Research Group, School of Public Health and Social Work; Queensland University of Technology, Brisbane, Queensland, 4059, Australia. Email address: [w2.hu@qut.edu.au](mailto:w2.hu@qut.edu.au).

**Table of Contents**

Table S1. Sensitivity analysis of adding ‘Holiday’ in the model.

Fig. S1. Köppen climate classification of Jinan and Qingdao, Shandong Province, China.

Fig. S2. Cumulative associations between heatwave days and T2DM death cases for adults and men (up to lag 2 weeks).

Fig. S3. Comparison between estimated data (e) and reported data (r) in a different surveillance report for T2DM death cases.

Fig. S4. Trends of heatwaves frequency in Jinan and Qingdao, 2013‒2019.

Table S1. Sensitivity analysis of adding ‘Holiday’ in the model.

|  | QAIC (Adults) | | QAIC (Elderly group) | |
| --- | --- | --- | --- | --- |
|  | Jinan | Qingdao | Jinan | Qingdao |
| Model | 612.0096 | 564.6291 | 618.1591 | 541.1158 |
| Model + Holiday | 615.7956 | 567.2829 | 618.4007 | 543.8714 |

Note: QAIC: quasi-Akaike information criterion;

Model: $\log\left[ E\left( Y_{t} \right) \right]=\beta_{0}+now+cb\left( {Tw}_{ld},df=3 \right)+ns\left( {PM}_{2.5},3 \right)+ns\left( time,df=4 \right)$.


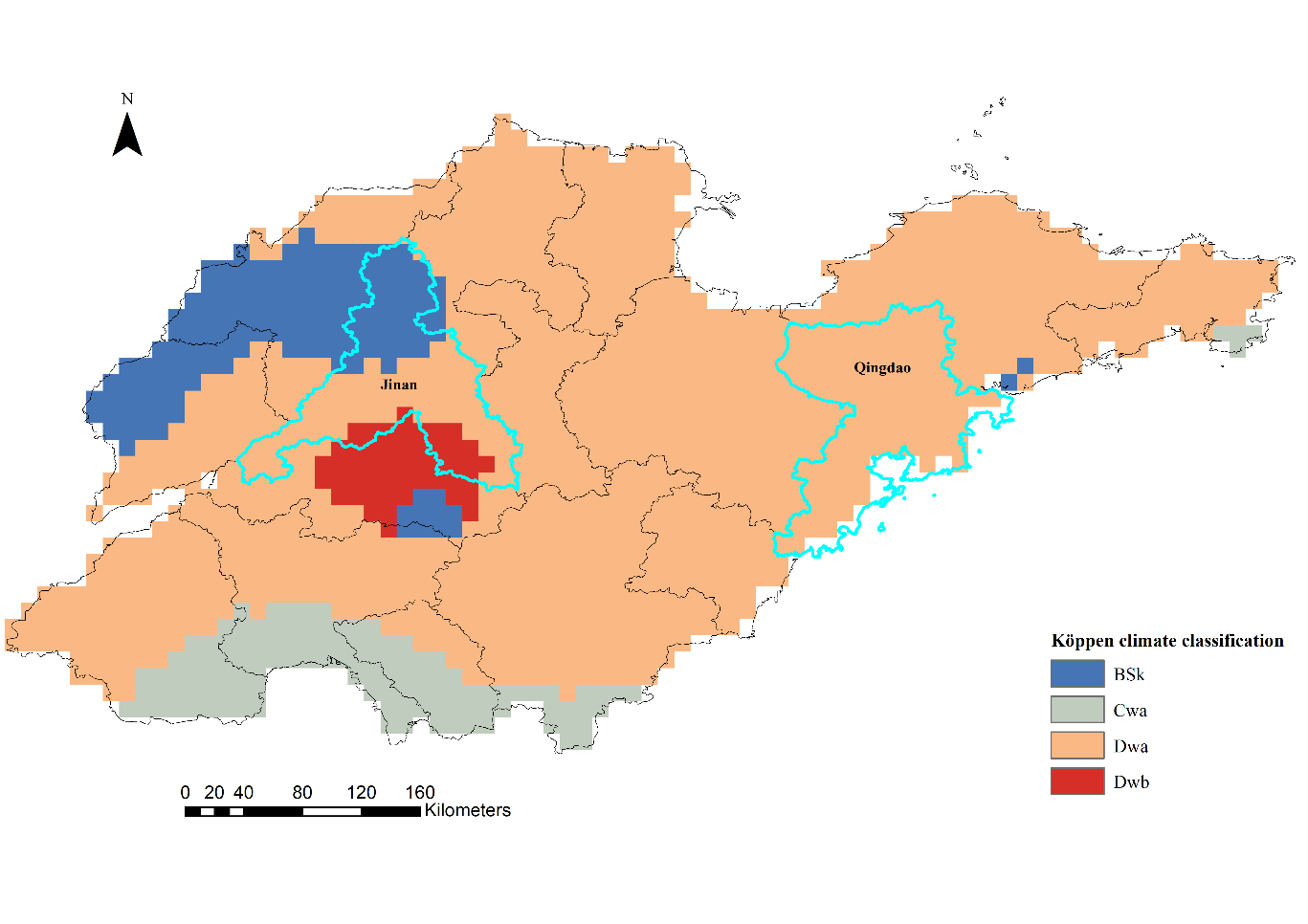


Note: BSk = Cold semi-arid climate; Dwa = Monsoon-influenced hot-summer humid continental climate; Dwb = Monsoon-influenced warm-summer humid continental climate; Cwa = Monsoon-influenced humid subtropical climate.

Fig. S1. Köppen climate classification of Jinan and Qingdao, Shandong Province, China.


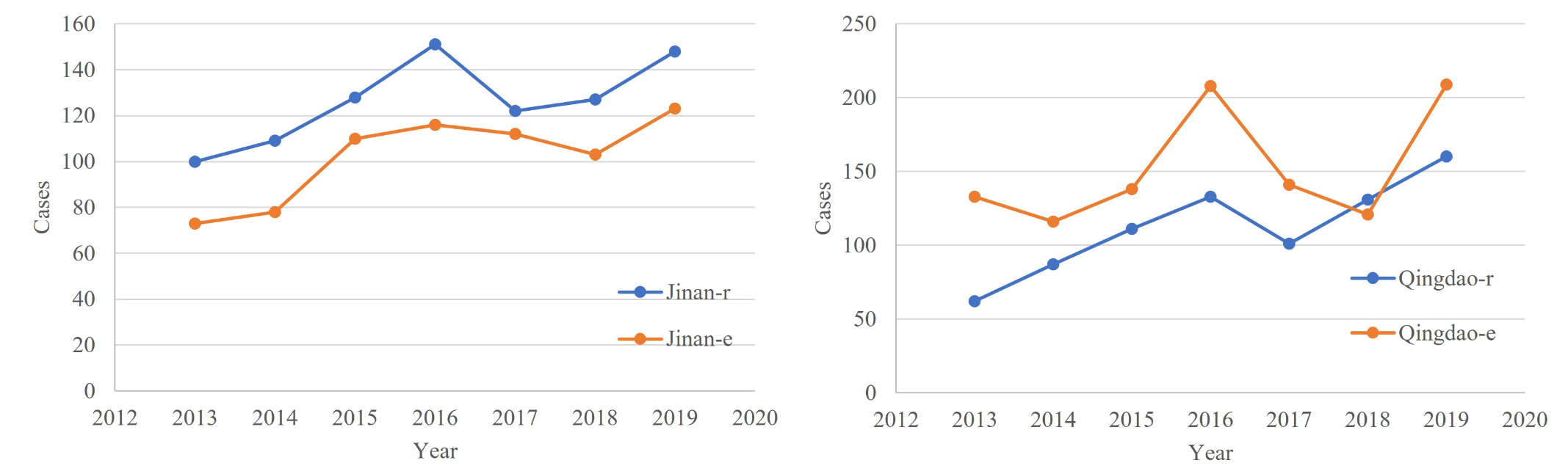


Figure S2. Comparison between estimated data (e) and reported data (r) in a different surveillance report for T2DM death cases.


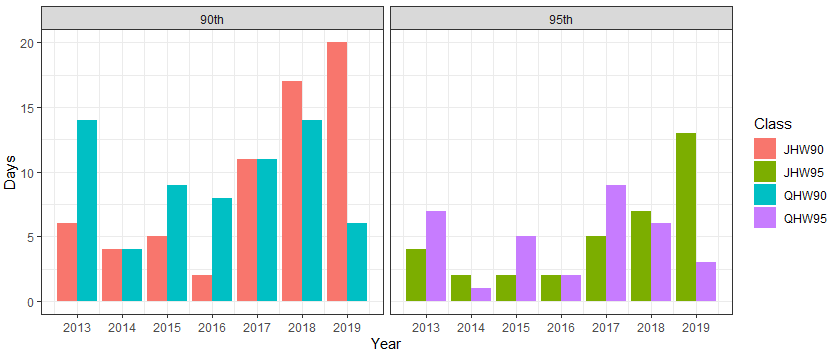


Fig. S4. Trends of heatwaves frequency in Jinan and Qingdao, 2013‒2019. (JHW90: daily mean temperature above 90^th^ heatwave in Jinan; JHW95: daily mean temperature above 95^th^ heatwave days in Jinan; QHW90: daily mean temperature above 90^th^ heatwave days in Qingdao; QHW95: daily mean temperature above 95^th^ heatwave days in Qingdao)
